# Two Cases of Newly Characterized *Neisseria* Species, Brazil

**DOI:** 10.3201/eid2602.190191

**Published:** 2020-02

**Authors:** Mustapha M. Mustapha, Ana Paula S. Lemos, Marissa P. Griffith, Daniel R. Evans, Ramon Marx, Elizabeth S.F. Coltro, Christian A. Siebra, Loeci Timm, Hamilton Ribeiro, Alessandro Monteiro, A. William Pasculle, Jane W. Marsh, Daria Van Tyne, Lee H. Harrison, Claudio T. Sacchi

**Affiliations:** University of Pittsburgh, Pittsburgh, Pennsylvania, USA (M.M. Mustapha, M.P. Griffith, D.R. Evans, A.W. Pasculle, J.W. Marsh, D. Van Tyne, L.H. Harrison);; Instituto Adolfo Lutz, São Paulo, Brazil (A.P.S. Lemos, C.T. Sacchi);; Hospital Nossa Senhora da Conceição, Porto Alegre, Brazil (R. Marx, A. Monteiro);; Hospital do Trabalhador/SESA, Curitiba, Brazil (E.S.F. Coltro);; Laboratório Central de Saúde Pública, Curitiba (C.A. Siebra);; Laboratório Central de Saúde Pública, Porto Alegre (L. Timm);; Paraná Department of Health, Piraquara, Brazil (H. Ribeiro)

**Keywords:** Neisseria brasiliensis, Neisseria meningititidis, meningococcal capsule, capsular switching, Neisseria species, whole genome sequencing, Brazil, surveillance, bacteria

## Abstract

We describe 2 human cases of infection with a new *Neisseria* species (putatively *N. brasiliensis*), 1 of which involved bacteremia. Genomic analyses found that both isolates were distinct strains of the same species, were closely related to *N. iguanae*, and contained a capsule synthesis operon similar to *N. meningitidis*.

*Neisseria* is a genus containing diverse organisms; most are rarely pathogenic. *N. meningitidis* and *N. gonorrhoeae* are the most clinically relevant species. The polysaccharide capsule is the most critical meningococcal virulence factor, a vaccine target, and the basis for classifying meningococci into serogroups (*1*). During routine laboratory-based public health surveillance in Brazil, we identified 2 cases of infection caused by a previously uncharacterized species of the *Neisseria* genus.

Clinicians reported 2 cases to the National Reference Laboratory, Adolfo Lutz Institute (IAL), São Paulo, Brazil. Case-patient 1 was a 64-year-old man from Rio Grande do Sul state, Brazil, who, in June 2016, had congestive heart failure with bilateral pulmonary infiltrates and pleural effusion on chest radiograph. Case-patient 2 was a 74-year-old woman with leprosy from Paraná state, Brazil, who, in February 2016, developed a polymicrobially infected ulcer of the left lower extremity. The 2 cases were separated in time and by >400 km and had no known epidemiologic link.

Overnight cultures of blood from case-patient 1 and ulcer exudate from case-patient 2 on brain–heart infusion agar containing 10% chocolate and horse blood at 37°C in the presence of 5% CO_2_ both revealed brownish colonies uncharacteristic of *N. meningitidis*. We identified both isolates (N.95-16, from case-patient 1, and N.177-16, from case-patient 2) as gram-negative glucose-fermenting diplococci with positive catalase and oxidase tests. The isolates fermented maltose, lactose, sucrose, and fructose but not mannose; they reduced nitrate and produced a starch-like polysaccharide detected with Gram’s iodine but did not produce DNase. Assessment by matrix-assisted laser desorption/ionization time-of-flight mass spectroscopy found no species match; the closest matches belonged to the *Neisseria* genus for both isolates. 

We performed serogrouping by slide agglutination (*2*) with polyclonal goat or horse antisera prepared at IAL against the *N. meningitidis* capsule groups (ABCEWXYZ), as described previously (*3*), and confirmed with real-time PCR (*4*). Isolate N.95-16 had strong agglutination against serogroup X and nonspecific agglutination against serogroups A, B, C, W, Y, E, and Z antisera; isolate N.177-16 had nonspecific agglutination against A, B, C, W, X, Y, E, and Z antisera. Meningococcal serogroup-specific real-time PCR identified isolate 1 as *N. meningitidis* capsular group X and isolate 2 as capsular group B.

We extracted genomic DNA from overnight cultures and performed library preparation and whole-genome sequencing using a combination of Illumina MiSeq (https://www.illumina.com) and Oxford Nanopore MinION (https://nanoporetech.com*)* technologies. Sequencing reads underwent hybrid assembly using Unicycler (*5*), which generated a high-quality draft assembly for isolate N.95-16 and a complete genome sequence for isolate N.177-16 (GenBank accession nos. WJXO00000000 and CP046027; PubMLST [https://pubmlst.org] identification 94178–94179). We performed species investigation by querying sequencing reads and assemblies against the GenBank and PubMLST reference databases (*6*). We aligned gene sequences corresponding to 53 conserved ribosomal MLST (rMLST) loci (*7*) across the *Neisseria* genus and constructed a maximum likelihood phylogenetic tree using RAxML with 1,000 bootstrap replicates (Figure, panel A). We calculated average nucleotide identity (ANI) using OrthoANI (*8*).

**Figure:**
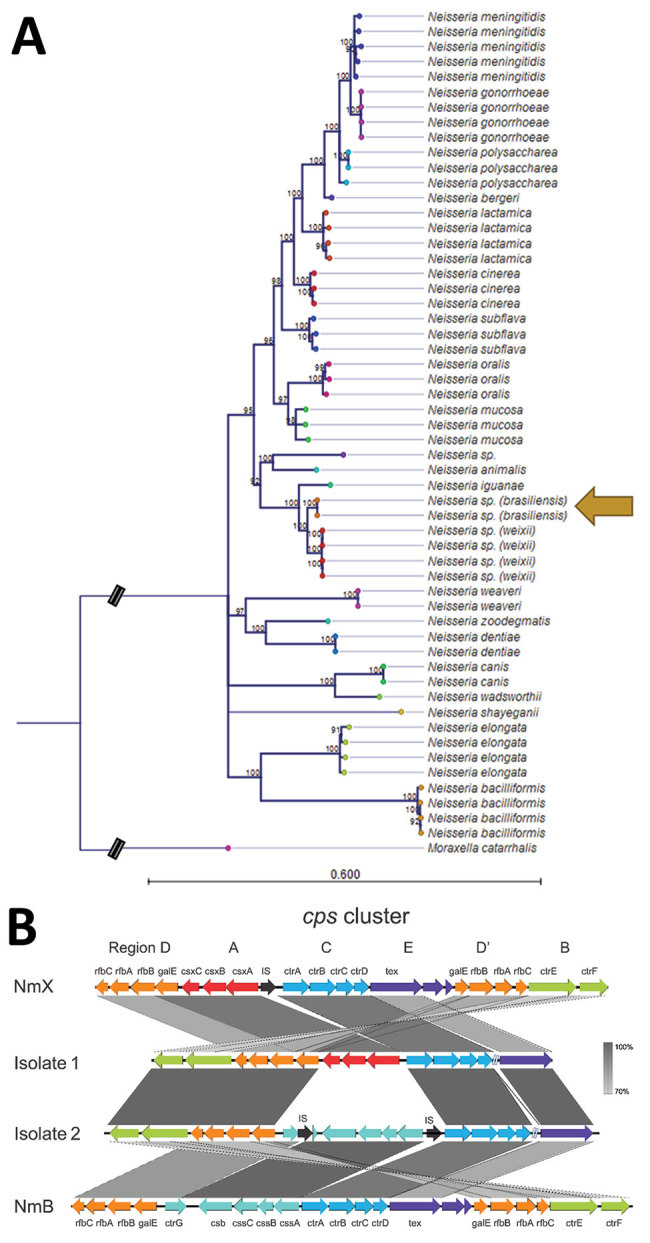
Analyses of newly characterized *Neisseria* species, Brazil. A) Maximum-likelihood phylogenetic tree of 53 aligned ribosomal multilocus sequence typing genes with 1,000 bootstrap replicates using *Moraxella catarrhalis* as an outgroup. Bootstrap support values <90% are not shown. Scale bar represents number of substitutions per site. B) *cps* sequences in isolates 1 (N.95-16) and 2 (N.177-16) relative to meningococcal reference genomes. Arrows represent genes, which are color coded by previously defined *cps* regions (*1*). Gray rectangles represent percentage nucleotide identity; darker shading indicates higher identity. The *tex* gene in both isolates is located >10 kb downstream of other *cps* genes. *cps*, capsule gene cluster; IS, insertion sequence; NmB, *N. meningitidis* serogroup B reference genome H44/76; NmX, *N. meningitidis* serogroup X reference sequence α388; IS, *transposase* gene.

Both isolate genomes were 2.5 Mb and had 49.2% guanine-cytosine (GC) content. ANI was 99.3% between the 2 genomes and <86% relative to all other *Neisseria* species. The closest genome matches were *N. iguanae* and the proposed *N. weixii*, isolated from the intestinal contents of a Tibetan Plateau pika (Figure, panel A) (PubMLST identification 56407–56409; GenBank accession no. CP023429). Both genomes shared identical rMLST profiles (rST 61343); the 4 proposed *N. weixii* genomes shared only 1–2 alleles of 53 rMLST loci with these isolate genomes, and *N. iguanae* shared no rMLST alleles. Both genomes contained an intact capsule gene cluster (*cps*) that was similar in gene organization and sequence identity to *N. meningitidis* (Figure, panel B). The *ctrA-cssA/csxA* promoter region was conserved in both isolates. However, both genomes contained only 1 copy of *galE*-*rfbCAB* (Region D), compared with 2 copies found in meningococcal reference genomes; the *tex* gene was located >10 kb outside *cps*, upstream from *ctrD* (Figure, panel B). The 2 isolates differed in their sequence of sialic acid biosynthesis genes within region A; isolate N.95-16 contained *csxABC* genes that shared 98% amino acid identity with the meningococcal serogroup X reference strain α388 (*1*), and isolate N.177-16 contained *cssABC*-*csb* genes that shared 99% amino acid identity with serogroup B reference strain H44/76 (Figure, panel B). The *cps* differences observed between the isolates were similar to the mosaic recombination pattern associated with meningococcal capsular switching (*9*). Taken together, the presence of *cps* genes sharing substantial similarity to meningococcal homologs suggests that both isolates have the potential to synthesize meningococcal-like capsules.

In summary, we describe 2 sporadic cases of a new *Neisseria* species (which we propose to name *Neisseria brasiliensis*), 1 of which also involved bacteremia. Both genomes contain an intact repertoire of genes for capsule synthesis, a key meningococcal virulence factor. The significance of capsule genes and potential capsule synthesis in nonmeningococcal *Neisseria* is unknown (*10*). Continued surveillance is required to establish the pathogenic potential and host range for this apparent new species.
